# Synthesis of mesoporous silica post-loaded by methyl eugenol as an environment-friendly slow-release bio pesticide

**DOI:** 10.1038/s41598-020-63015-6

**Published:** 2020-04-09

**Authors:** Huayao Chen, Lishen Chen, Zhichuan Shen, Hongjun Zhou, Li Hao, Hua Xu, Xinhua Zhou

**Affiliations:** 1 School of Chemistry and Chemical Engineering, Zhongkai University of Agriculture and Engineering, Guangzhou, P.R. China; 2 Key Laboratory of Agricultural Green Fine Chemicals of Guangdong Higher Education Institution, Guangzhou, P.R. China

**Keywords:** Drug delivery, Drug delivery, Nanopores

## Abstract

Salicylaldimine, furfuralimine and benzaldehyde imine were adopted to modify mesoporous silica (MCM) respectively denoted as Sal-MCM, Fur-MCM and Ben-MCM before loading methyl eugenol (Me) for pesticide delivery. Me was adsorbed by Schiff base mesoporous silica without destructing regular hexagonal pore structure verified by the characterization results. DSC result implied that Me in amorphous state which was distributed in the pores of the mesoporous silica. The loading content of Me-Sal-MCM, Me-Fur-MCM and Me-Ben-MCM 67.89%, 73.34% and 73.84% which was higher than Me-MCM without modification (67.35%).Because the electrostatic interaction and π-π interaction between Schiff base and Me strengthened the adsorption capacity of the carrier. And the electrostatic interaction played a more important role in interaction between Me and Schiff base modified mesoporous silica. As a result, Schiff base modified sustained release system also has significantly longer sustained release time with a sequence of Me-Sal-MCM > Me-Ben-MCM > Me-Fur-MCM in release speed in negative correlation with the electric potential sequence. The behaviors of their sustained release performance can be fitted by First order kinetic model before Schiff base modification. After modification, their sustained release behaviors were consistent with Korsmeyer-Peppas equation with non-Fickian diffusion mechanism indicating that main impact on the release process after modification was no longer mainly controlled by the difference of the concentration. Finally, the highest lure rate of the modified MCM (Me-Fur-MCM) equals to the 73% of the pure Me due to its highest BET surface area and strongest interaction with Me among the three Schiff base modified samples. Therefore, the environment-friendly slow-release bio pesticide with long service life was prepared to reduce the damage on the environment caused by pesticide.

## Introduction

Worldwide, more effective, safer detection and control systems for invasive insect pest management become more and more important. Phenyl propanoids are attractive to numerous species of Dacinae fruit flies. Among them, methyl eugenol (Me) (4-allyl-1,2-dimethoxybenzene) is a widely distributed natural plant product and originated from more than two hundred plant species found mainly in the tropical area such as anise, nutmeg, basil, blackberry essence, bananas, citrus and so on^1^. Me is also used as a flavoring in many foods due to its low toxicity^2^. For example, clove oil contains approximately 15% Me and is generally recognized as a safe (GRAS) compound by the U.S. Food and Drug Administration as a food additive^3^. Other benefits used as aromatherapy, fragrance in the perfume^4^, cosmetics^5^, toiletries and detergents^6^. Methyl eugenol has been used as an anesthetic^7^ even as towing insects in combination with insecticides^8^.

In southern China, agricultural production and the product export trade were severely damaged by Bactrocera dorsalis^9^. The traditional method of controlling this pest is the application of insecticide cover sprays. However, the usage of pesticide would affect the growth of the fruits and the pesticide remained on the fruits would also damage the health of human beings^10^. Therefore, Me is currently the most commonly used tephritid male attractant lure. Initially, wild Bactrocera dorsalis males were successfully controlled with Me-baited traps^11,12^. Nevertheless, Me discomposes quickly in soil or water at room temperature with a short half-life in air which was around 5 hours according to previous report^13^.

Recently, replacement of liquid Me and insecticides with solid formulations has drawn great interests for convenience and worker safety^14^. In previous work, the pH responsive Schiff base modified mesoporous silica was prepared for chlorpyrifos^15^, triazolone^16^ and avermectin^17^ delivery which showed a high performance for adsorption and sustained release. In order to improve Me stability and prolong its service life, we proposed the Schiff base modified mesoporous silica sustained release system for essential oil sustained release^18^. The interaction of mesoporous silica with Me was anticipated to be strengthened by π-π interaction between the benzene ring from Me and aromatics from Schiff base and electrostatic interaction between Schiff base with positive charge and Me with negative charge. In our recent work^19^, mesoporous silica was modified by salicylaldimine, furfuralimine, and benzaldehyde imine, respectively, delivery by one-step method which showed smooth sustained release curves. However, the loading content of Me was relatively low (less than 25%) properly due to the structure defects caused by one-step method. What’s more, the templates remained can’t be removed in one-step method which would significantly reduce its BET surface.

Based on the researches above, the self-made salicylaldimine, furfuralimine and benzaldehyde imine were used to modify mesoporous silica by grafted with coupling agent 3-aminopropyltriethyloxy silane (APTES). Different from previous study^19^, Me was post-loaded in the mesoporous after removing the templates in the pores which eliminated Me affection on the co-condensation process and resulted in much higher loading content. The relationship between the host/guest interaction and sustained release mechanism was also studied.

## Materials and Methods

### Chemicals

Cetyl trimethyl ammonium bromide (CTAB), tetraethyl orthosilicate (TEOS), ethanol, dichloromethane, ammonia, sodium hydroxide, hydrochloride were obtained from Tianjin Damao Chemical Reagents. 3-aminopropyltriethyloxy silane (APTES), salicylaldehyde, furaldehyde and benzoaldehyde were obtained from Aladdin. And methyl eugenol (Macklin Co., Ltd.) was also used in this work. All chemicals were analytical grade and used as received without any further purification.

### Preparation of salicylaldimine, furfuralimine and benzaldehyde imine grafted on APTES

According to the literature^19,20^, 4.42 g of APTES, 2.44 g of salicylaldehyde and 100 mL of ethanol were added into a flask and refluxed at 95 °C for 3 h. Ethanol was removed through rotary evaporation. 20 mL of dichloromethane was added, then the products washed with deionized water 3 times. The organic layer was extracted and standing for 12 h. Then the product was filtered to remove dichloromethane to attain salicylaldimine. The furfuralimine and benzaldehyde imine were prepared though the same method illustrated above while salicylaldehyde was replaced by furldehyde and benzaldehyde respectively.

### Preparation of MCM, Sal-MCM, Fur-MCM and Ben-MCM

According to previous research^15^, co-condensation method was adopted to prepare salicylaldehyde modified mesoporous silica (Sal-MCM. 1.0 g of CTAB, 100 mL deionized water and 70 mL of ammonia were added to the flask to be dissolved at 60 °C with stirring. And 5 g of TEOS was added to the solution dropwise. 1 hour later, 1 g of as synthesized salicylaldimine was added and kept on reacting for 6 h before being crystalized at room temperature, filtered, washed and dried. Finally, the template was removed by ethanol to attain Sal-MCM Using this approach, the final products Fur-MCM and Ben-MCM (i.e salicylaldimine replaced by furfuralimine and benzaldehyde imine, respectively) were prepared. In comparision, MCM was prepared as mentioned above without adding salicylaldimine.

### Loading of methyl eugenol

According to previous study^19^, the supported Me was prepared via impregnation. The mesoporous silica were activated under vacuum at 80 °C for 12 h. And 0.25 g of samples was immersed in 250 mg Me at room temperature for 1 h, then filtered and dried. The samples obtained were denoted as Me-Sal-MCM, Me-Fur-MCM and Me-Ben-MCM respectively according to the different carrier. And the loading content was calculated through TG method.

### Measurements

The small angel X-ray diffraction (SAXD) were performed using a Bruker AXS D8 X-ray diffractometer (Bruker, Germany) with Cu radiation (λ = 1.5418 Å) and a graphite monochromator at 25 °C, 40 kV, and 30 mA. The measurements were scanned at 2°/min (angular range 2θ = 0.5~10°) in 0.02° step size. FTIR spectra were recorded in the region 4000–400 cm^−1^ by a Spectrum100 Fourier infrared spectrometer (PerkinElmer, USA) using the KBr squash technique. The gold particles were sprayed on the surface of samples under protection of N_2_ and the samples were characterized by an S4800 scanning electron microscope (Hitachi, Japan) to observe the surface topography. TEM observation was conducted on a FEI Tecnai G2 F20 transmission election microscope (Thermo Fisher Scientific, USA). BET surface area of samples was determined by N_2_ adsorption isotherms at 77 K, operated on Quadrasorb SI adsorption equipment (Quantachrome, USA). The samples were degassed at 200 °C for 12 h in vacuum before N_2_ adsorption experiment. A Q200 differential scanning calorimeter (TA Instruments, USA) was used to conduct differential scanning calorimetry and detect the crystalline degree of the mesoporous silica in the particles over a heating range of 0~200 °C and a heating rate of 10 °C/min under the protection of N_2_ (flow rate 50 mL/min). Thermal gravity (TG) measurements of these samples were carried out on an SDT-Q600 thermogravimetric analyzer (TA Instruments, USA) analyze the heat stability of particles over the heating range of 40~600 °C under the condition of N_2_ flow rate 50 mL/min and heating rate 10 °C. X-ray photoelectron spectra (XPS) were recorded on a ESCALAB250XI spectrometer (Thermo Fisher Scientific, USA) under a vacuum of ~2 × 10^−7^ Pa. Charging effects were corrected by adjusting the main C 1 s peak to a position of 284.8 eV. The zeta potential of the samples was investigated with a Zetasizer Nano ZS (Malvern, UK) in water at pH 7 through ultrasonic dispersion.

### Sustained release performance test

According to previous study^19^, the performance of sustained release Me particles was tested by TG method which has been adapted in our previous research^19^. Drug-loaded particles (3–5 mg) were weighed and placed in a Al_2_O_3_ crucible of SDT-Q600 thermogravimetric analyzer in 60 °C for 10 h, and the flow rate of N_2_ was 50 mL/min. At intervals of (*t*), and the cumulative release amount of Me was calculated as *R*_*i*_*. A t–R*_*i*_ curve was drawn to study the release kinetics of Me.

### Kinetic study

Sustained release curves were fitted by the first-order (Eq. (1)), Higuchi (Eq. (2)), Korsmeyer-Peppas (Eq. (3)), Quadratic (Eq. (4)), Logistic (Eq. (5)) and Weibull model (Eq. (6)). The linearized forms of these equations are expressed as below^21–23^:

1 $$y={K}_{2}[1-\exp (-{K}_{1}t)]$$

2 $$y={K}_{1}{t}^{1/2}$$

3 $$y={K}_{2}{t}^{{K}_{1}}$$

4 $$y=100\,({K}_{1}{t}^{2}+{K}_{2}t)$$

5 $$y={K}_{3}/\{1+\exp [-{K}_{2}(t-{K}_{1})]\}$$

6 $$y=1-\exp [(-{t}^{{K}_{2}})/{K}_{1}]$$

where *y* is the experimental accumulated rate of Me (%) at time *t*, respectively. *K* is the rate constant for the kinectic model. The correlation coefficient (R^2^) and the release exponent (n) were used to determine the best-fit kinetic model and the mechanism of the drug release.

### Attraction of bactrocera dorsalis test

According to the literature^8,19^, A certain among of male Bactrocera Dorsalis with sexual maturation were transferred to a 45 cm × 45 cm × 45 cm cage. Steiner trap contained 100 mg sample was placed in the center of the cage. And the number of Bactrocera Dorsalis trapped in the Steiner trap was recorded to calculate the lure rate.

## Results and discussion

### Structure characterization

**Figure 1:**
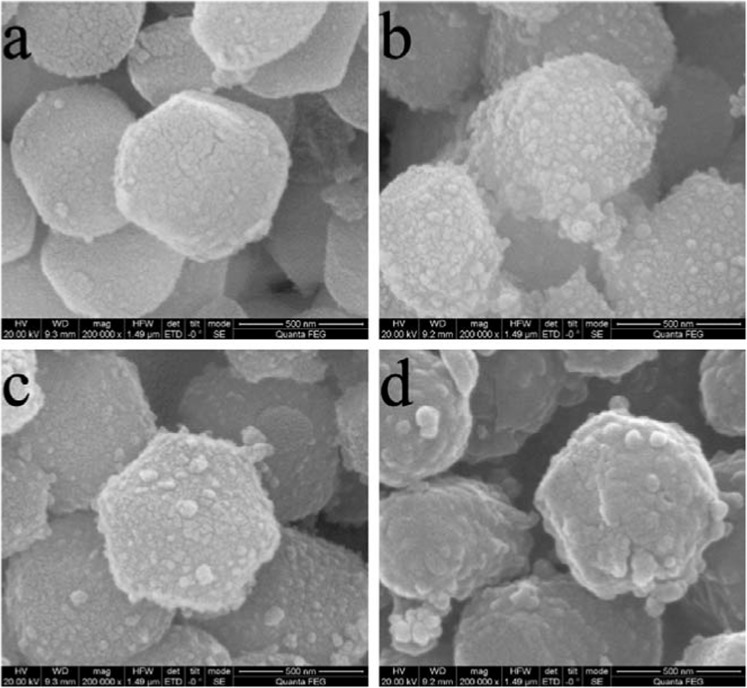
SEM images of MCM (**a**), Sal-MCM (**b**), Fur-MCM (**c**) and Ben-MCM (**d**).

**Figure 2:**
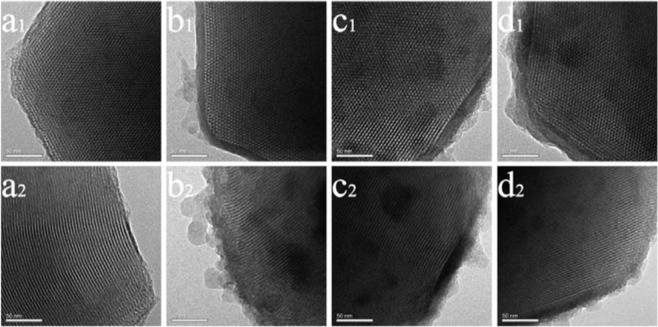
TEM images of MCM (**a**_**1**_**,a**_**2**_), Sal-MCM (**b**_**1**_**,b**_**2**_), Fur-MCM (**c**_**1**_**,c**_**2**_) and Ben-MCM (**d**_**1**_**,d**_**2**_).

SEM and TEM images of MCM samples before and after modification were depicted in Figs. 1 and 2. The regular hexagonal pore structure and the structure integrity from MCM^24^ was well-maintained^25^ after modification and the orderings of the (100) and (110) was not affected by Schiff base co-condensation. The particle size of the four samples were 833 nm, 789 nm, 701 nm and 763 nm respectively for MCM, Sal-MCM, Fur-MCM and Ben-MCM measured from the SEM images. And the surface of MCM become rough with small particles formed on the surface as the SEM images shown in Fig. 1 after modification. Also a layered of shell structure appeared on the surface as the TEM images from Fig. 2 showed due to the precipitation of silane coupling agent^26^.

**Figure 3:**
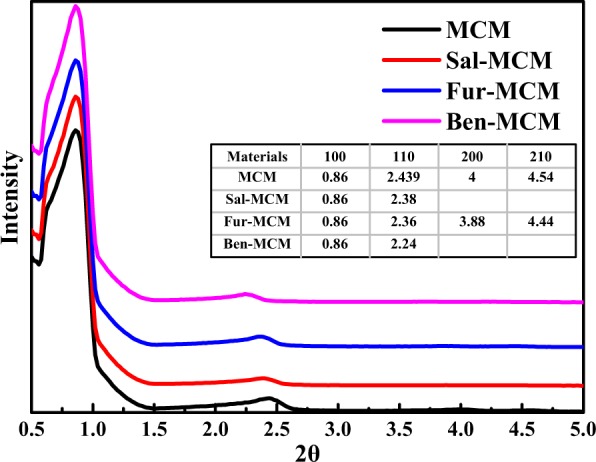
SAXD Patterns of MCM, Sal-MCM, Fur-MCM and Ben-MCM.

Figure 3 depicted the SAXD patterns of the samples. Four characteristic peaks at 0.860°, 2.439°, 4.000° and 4.540° were ascribed to (100), (110), (200) and (210) crystal faces respectively. And the crystal faces of (100) and (110) were also shown in the TEM images as illustrated in Fig. 2 representing for the regular hexagonal pore structure in accordance with SEM and TEM images. However, the (100) diffraction of the modification samples was shown to further shift toward smaller 2θ value due to the block of the pore by Schiff bass. The 2θ value shift of (200) and (210) crystal faces also happened for Fur-MCM. What’s more, the peaks ascribed to (200) and (210) crystal faces disappeared for Sal-MCM and Ben-MCM which proved that benzene ring was introduced to the system and decreased its degree of orderliness^27^.

**Figure 4:**
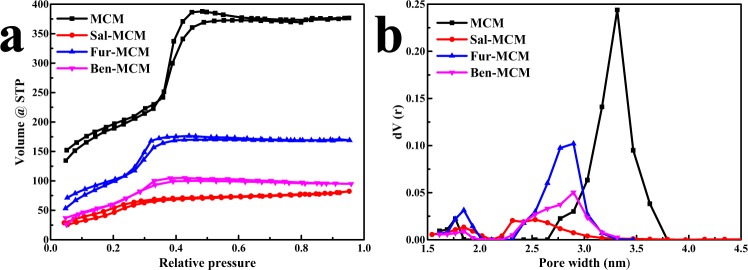
N_2_ adsorption/ desorption isotherms (**a**) and pore size distribution (**b**) of MCM, Sal-MCM, Fur-MCM and Ben-MCM.

As shown in Fig. 4a, the N_2_ adsorption/desorption isotherms of MCM, Sal-MCM, Fur-MCM and Ben-MCM belong to Langmuir IV (the slope of it was decreasing) which indicated that their pore size was relatively small and also confirmed by the pore size distribution results calculated by DFT method^28^ as shown in Fig. 4b. The N_2_ adsorption/desorption isotherms in Fig. 4 do not overlap at relative pressures <0.2 were due to N_2_ chemisorbed by silanol on the surface of the pores which confirmed by our previous researches^18,26^. The rapid shift of N_2_ adsorption isotherms caused by capillary condensation inside the pore for MCM disappeared after modification. And the BET surface, pore size and pore volume decreased after modification due to the pore block by Schiff base. And the BET surface of Sal-MCM, Fur-MCM and Ben-MCM after modification were 225.5 m^2^/g, 413.4 m^2^/g and 274.8 m^2^/g respectively which illustrated the difference in the degree of pore block by three different Schiff base as shown in Table 1 which was much higher than our previous researches (<200 m^2^/g) due to the templates removed from the samples. Fur-MCM had the highest BET surface among and largest pore size and pore volume among the Schiff base modified MCM indicating that the surface of the pores from MCM were homogeneously grafted by furalimine probably due to its strongest interaction towards to the substrate.

**Table 1:** The pore structural parameters of MCM, Sal-MCM, Fur-MCM and Ben-MCM.

Sample	BET surface (m^2^/g)	Pore size (nm)	Pore volumn (cm^3^/g)
MCM	681.8	3.315	0.573
Sal-MCM	225.5	2.531	0.114
Fur-MCM	413.4	2.897	0.261
Ben-MCM	274.8	2.897	0.156

The particle diameters and Zeta potential studies of MCM, Sal-MCM, Fur-MCM and Ben-MCM were investigated at pH 7 as listed in Table 2. The particle size calculated by DLS method was bigger than the particle size shown in SEM images due to the agglomeration and solvation effect since DLS measurements are conducted in solution according with previous research^29^. The particle size of Sal-MCM, Fur-MCM and Ben-MCM decreased after modification. The decrease in particle size was caused by the effect of Schiff base on TEOS hydrolysis during the condensation. And the sufficient Si-OH groups on the surface MCM before modification made it Zeta potential negative. After modification, Zeta potential of the samples shifted to be positive which confirmed the introduction of Schiff base into the mesoporous samples. Among them, Fur-MCM has the highest Zeta potential leading to its strongest electrostatic interaction with Me in consistent with BET results.

**Table 2:** The Particle diameters and Zeta potential of MCM, Sal-MCM, Fur-MCM and Ben-MCM.

Sample	SEM particle size (nm)	DLS particle size (nm)	PDI*	Zeta potential (mV)
Me	—	—	—	−38.82
MCM	833 ± 11	951.79	0.237	−20.16
Sal-MCM	789 ± 12	917.39	0.242	15.77
Fur-MCM	701 ± 12	740.52	0.362	29.89
Ben-MCM	763 ± 12	671.44	0.371	25.70

^*^PDI: Polydispersity Index.

**Figure 5:**
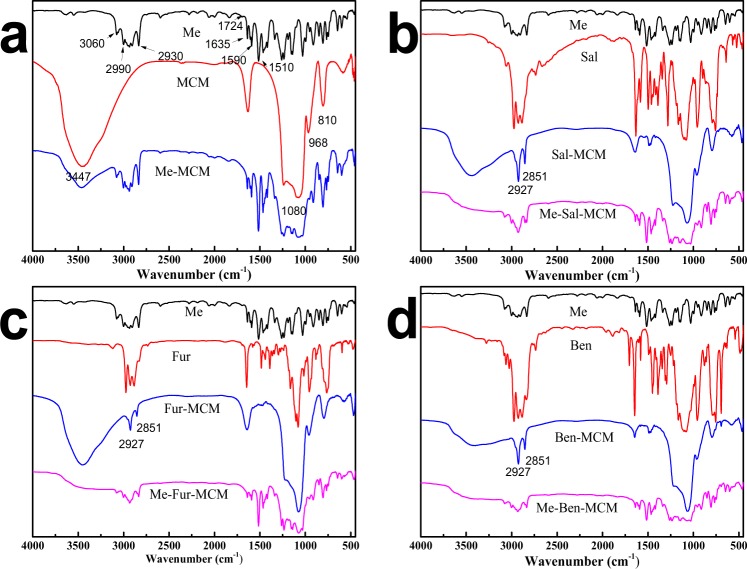
FTIR spectra of Me, Sal, Fur, Ben, MCM, Sal-MCM, Fur-MCM, Ben-MCM and Me-MCM, Me-Sal-MCM, Me-Fur-MCM, Me-Ben-MCM.

The FTIR spectra of MCM, Sal-MCM, Fur-MCM and Ben-MCM before and after loading Me were depicted in Fig. 5. Two bands appeared in 3447 cm^−1^ and 968 cm^−1^ for MCM ascribed to stretching and bending vibration of Si-OH respectively. 1080 cm^−1^ and 810 cm^−1^ were attributed to the characteristic peaks of Si-O-Si on the SiO_2_ framework^30^. Comparing to MCM, two new bands appeared at 2927 cm^−1^ and 2851 cm^−1^ belonging to the symmetric and nonsymmetrical C-H stretching vibration bands from amino group for Schiff base which indicated the Schiff base was successfully grafted to MCM. After loading Me, the characteristic peaks of Me located at 3060, 2990, 2930, 1635, 1590 and 1510 cm^−1^ which proved that the Me was successfully adsorbed by mesoporous silica^31^. The IR spectra of Me showed a peak at 1724 cm^−1^ for carbonyl group which was gradually disappearing after loaded on mesoporous silica due to the interaction with MCM substrate. Intensity of peaks between 2800 and 3100 cm^−1^ ascribed to the C–H stretching band was increased significantly showing that Me was successfully impregnated with mesoporous silica.

**Figure 6:**
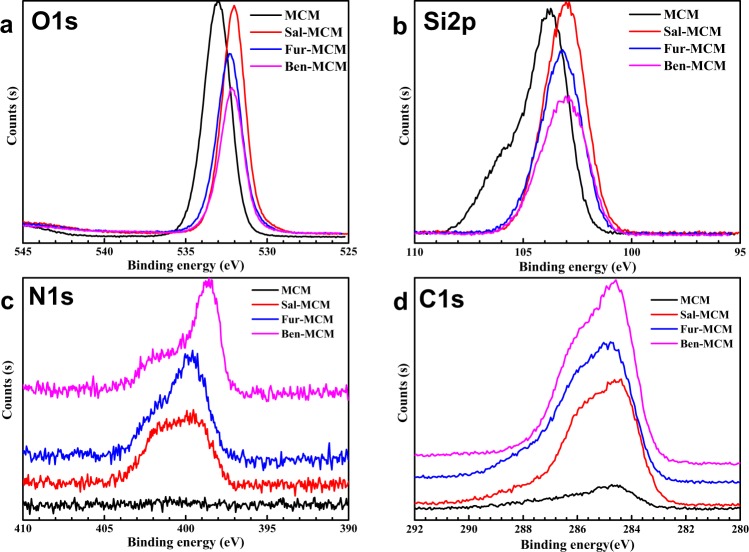
XPS of O1s (**a**), Si 2p (**b**), N 1 s (**c**), C 1s (**d**) in MCM, Sal-MCM, Fur-MCM and Ben-MCM.

The surface elements chemical states were observed with XPS analysis as shown in Fig. 6 and Table 3. The binding energy (BE) negative shift of Si 2p as shown in Fig. 6a,b were observed after Schiff base modification due to the reaction between Si-OC_2_H_5_ from Schiff base and Si-OH from MCM^15^. The N 1 s peaks as shown in Fig. 6c were observed after Schiff base modification, with a BE value of 399.98 eV, 399.74 eV and 398.65 eV for Sal-MCM, Fur-MCM and Ben-MCM. In the meanwhile, The C 1 s peaks as shown in Fig. 6d also appeared with BE value of 285.73 eV, 285.68 eV and 286.68 eV respectively for Sal-MCM, Fur-MCM and Ben-MCM which convinced the introduction of Schiff base.

**Table 3:** Binding energy (BE) and the atomic percentage of MCM, Sal-MCM, Fur-MCM and Ben-MCM.

Sample	BE/eV				Atomic/%			
	C1s	N1s	Si2p	O1s	N1s	Si2p	O1s	C1s
MCM	—	—	103.80	533.04	—	30.22	62.84	—
Sal-MCM	284.80	399.98	103.02	532.05	3.51	22.13	46.31	28.05
Fur-MCM	285.04	399.74	103.21	532.30	4.42	19.07	42.37	34.13
Ben-MCM	284.82	398.65	103.06	532.15	4.67	17.42	35.36	42.55

**Figure 7:**
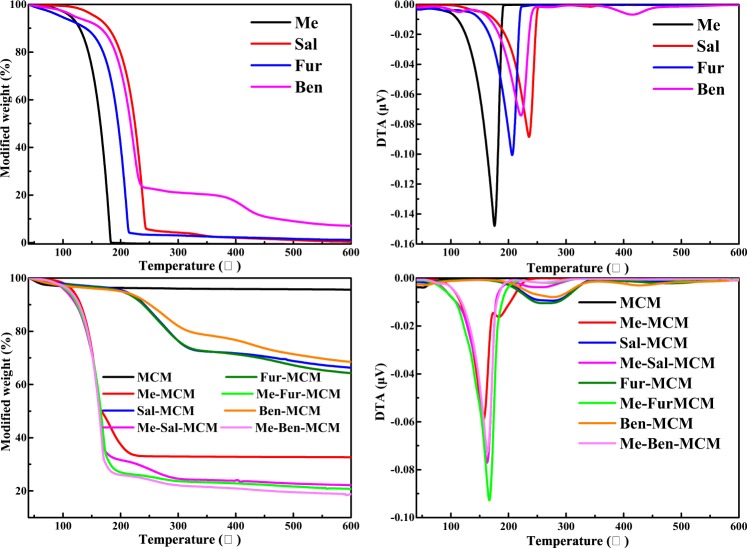
TG and DTG curves of Me, Sal, Fur, Ben, MCM, Me-MCM, Sal-MCM, Me-Sal-MCM, Fur-MCM, Me-Fur-MCM, Ben-MCM, and Me-Ben-MCM.

**Figure 8:**
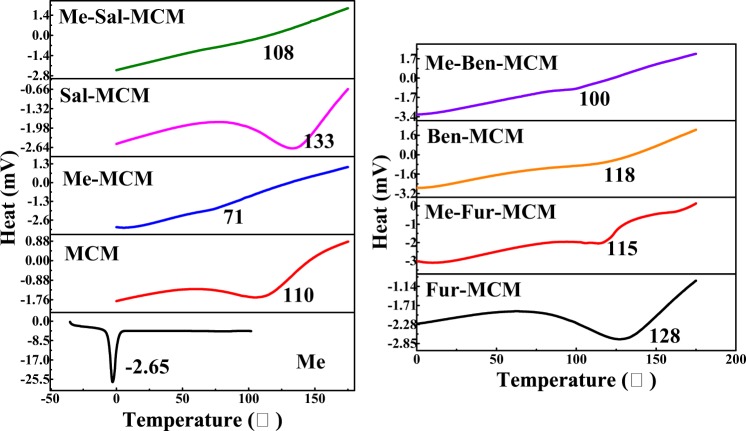
DSC curves of MCM, Me, Me-MCM, Sal-MCM, Me-Sal-MCM, Fur-MCM, Me-Fur-MCM, Ben-MCM, and Me-Ben-MCM.

Thermogravimetric analysis (TG) was used to investigate the thermal stability. As shown in Fig. 7, the loss in mass for MCM below 100 °C was caused the evaporation of water adsorbed by the samples^32^ in accordance with DSC results as shown in Fig. 8. The significant loss in mass occurred within the temperature range 160–350 °C which was caused by the decomposition of Schiff base on the mesoporous silica for Sal-MCM and Fur-MCM. While for Ben, 20% mass still existed even above 350 °C indicating that the mass loss was caused by the carbonization of Ben. After loading Me, the loss percentage in mass for Me-MCM, Me-Sal-MCM, Me-Fur-MCM and Me-Ben-MCM were apparently higher than Me-MCM without Schiff base modification due to the higher loading content of Me and the decomposition or carbonization of Schiff base. The loading content of Me-Sal-MCM, Me-Fur-MCM and Me-Ben-MCM were calculated from loss percentage within the temperature range 160~350 °C subtracting the mass of Schiff base. The loading content of Me-Sal-MCM, Me-Fur-MCM and Me-Ben-MCM 67.89%, 73.34% and 73.84% which was higher than Me-MCM without modification (67.35%) and much higher than the mesoporous silica prepared by our recent reported^19^ (<25%)

And the DSC thermograms of MCM, Me-MCM, Sal-MCM, Me-Sal-MCM, Fur-MCM, Me-Fur-MCM, Ben-MCM and Me-Ben-MCM were shown in Fig. 8. The fusion peaks of Schiff base modified mesoporous silica had a significant shift from 110 °C to 133 °C, 128 °C and 118 °C for Sal-MCM Fur-MCM and Ben-MCM comparing with MCM without modification which convinced the interaction between Schiff base and mesoporous silica in accordance with the XPS results. Another shift of the fusion peak happened after loading Me from 110 °C, 133 °C, 128 °C and 118 °C to 71 °C, 108 °C, 115 °C and 100 °C for Me-MCM, Me-Sal-MCM, Me-Fur-MCM and Me-Ben-MCM respectively due to the induction effect from Me. However, the disappearance of Me fusion peak proved that the Me is distributed homogeneously in amorphous state in the pores of the mesoporous silica.

### Sustained release test

**Figure 9:**
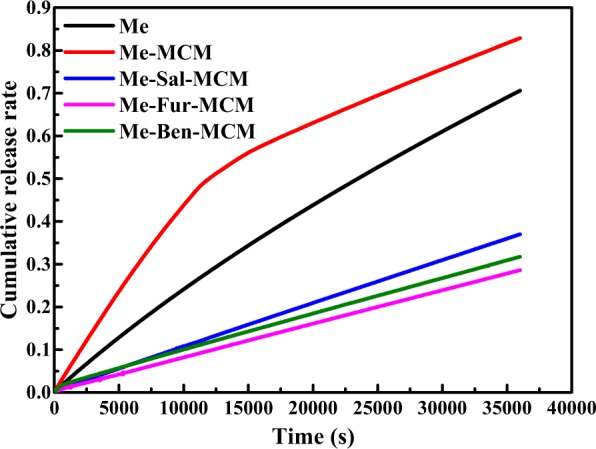
Sustained release curves of Me, Me-MCM, Me-Sal-MCM, Me-Fur-MCM and Me-Ben-MCM.

Figure 9 showed the sustained release performance of Me, Me-MCM, Me-Sal-MCM, Me-Fur-MCM and Me-Ben-MCM. The 50% accumulated release rates were attained at 389 min and 196 min for Me and Me-MCM. The rapid release for Me-MCM is probably due to the weak interaction between Me and MCM before modification. In comparison, the accumulated release rate of Me-Sal-MCM, Me-Fur-MCM and Me-Ben-MCM was less than 50% in 600 min which indicated that Schiff base modification could prevent the volatilization of Me and prolonged its service life. This slow release may be ascribed to the Schiff base modification which strengthened the interaction with mesoporous silica by π-π interaction between the benzene ring from Me and aromatics from Schiff base and electrostatic interaction between Schiff base with positive charge and Me with negative charge. And Fig. 9 also depicts the sustained release curves with a sequence of Me-Sal-MCM > Me-Ben-MCM > Me-Fur-MCM in release speed in negative correlation with the electric potential sequence shown in Table 2 for the three samples indicating that the electrostatic interaction played a more important role in interaction between Me and Schiff base modified mesoporous silica. And the ultimate accumulated release rate for Me, Me-MCM, Me-Sal-MCM, Me-Fur-MCM and Me-Ben-MCM were 70.82%, 82.88%, 48.59%, 56.63% and 37.21% respectively in 10 hours.

### Kinetics study

To further understand the sustained release mechanism, the data of sustained release of Me and Me loaded in various mesoporous silica were fitted to different kinetic models (Table 4). The sustained release curves of Me and Me-MCM were in accordance with first-order kinetic equation proving the barrier-free diffusion of the drug. While Me-Sal-MCM, Me-Fur-MCM and Me-Ben-MCM were in consistent with Korsmeryer-Pappas kinetic equation. The diffusion coefficients *K*_*1*_ of the fitting equations were between 0.45~1 controlled by a non-Fickian diffusion mechanism^33,34^. The sustained release behavior of MCM changed after modification because the difference of the concentration was no longer the main factor on controlling the release performance due to the strengthened interaction between Me and the substrate. Therefore, the service life of Me was prolonged.

**Table 4:** Fitting results for release curves of Me, Me-MCM, Me-Sal-MCM, Me-Fur-MCM and Me-Ben-MCM.

Release model	Materials	Parameters			
		*K*_1_	*K*_2_	*K*_3_	*R*^2^
First-order model	Me	1.6867 * 10^−5^	1.5388	—	0.9998
	Me-MSN	6.4813 * 10^−5^	0.8856	—	0.9967
	Me-Sal-MSN	3.4589 * 10^−6^	3.1453	—	0.9999
	Me-Fur-MSN	2.3894 * 10^−6^	3.4595	—	0.9999
	Me-Ben-MSN	1.0168 * 10^−5^	1.0188	—	0.9977
Higuchi model	Me	0.0032	—	—	0.8965
	Me-MSN	0.0044	—	—	0.9715
	Me-Sal-MSN	0.0016	—	—	0.8537
	Me-Fur-MSN	0.0012	—	—	0.8498
	Me-Ben-MSN	0.0014			0.8804
Korsmeyer-Peppas model	Me	0.8437	1.0203 * 10^−4^	—	0.9996
	Me-MSN	0.5699	0.0022	—	0.9802
	Me-Sal-MSN	0.9618	1.5311 * 10^−5^	—	0.9999
	Me-Fur-MSN	0.9725	1.0590 * 10^−5^	—	0.9999
	Me-Ben-MSN	0.8878	2.8305 * 10^−5^	—	0.9991
Quadratic model	Me	−1.6677 * 10^−12^	2.5395 * 10^−7^	—	0.9997
	Me-MSN	−7.1676 * 10^−12^	4.7091 * 10^−7^	—	0.9855
	Me-Sal-MSN	−1.7682 * 10^−13^	1.0865 * 10^−7^	—	0.9999
	Me-Fur-MSN	−9.4523 * 10^−14^	8.2606 * 10^−8^	—	0.9998
	Me-Ben-MSN	−4.3702 * 10^−13^	1.0245 * 10^−7^	—	0.9976
Logistic model	Me	—	—	—	—
	Me-MSN	9587.6467	1.8295 * 10^−4^	0.7670	0.9740
	Me-Sal-MSN	19117.9444	1.2175 * 10^−4^	0.3983	0.9914
	Me-Fur-MSN	19319.6768	1.2142 * 10^−4^	0.3094	0.9922
	Me-Ben-MSN	18804.2997	1.1392 * 10^−4^	0.3471	0.9926
Weibull model	Me	—	—	—	—
	Me-MSN	6924.9184	0.8938	—	0.9957
	Me-Sal-MSN	193080.1479	1.0843	—	0.9992
	Me-Fur-MSN	211409.7915	1.0636	—	0.9995
	Me-Ben-MSN	80090.7240	0.9818	—	0.9978

### Attraction of bactrocera dorsalis

**Figure 10:**
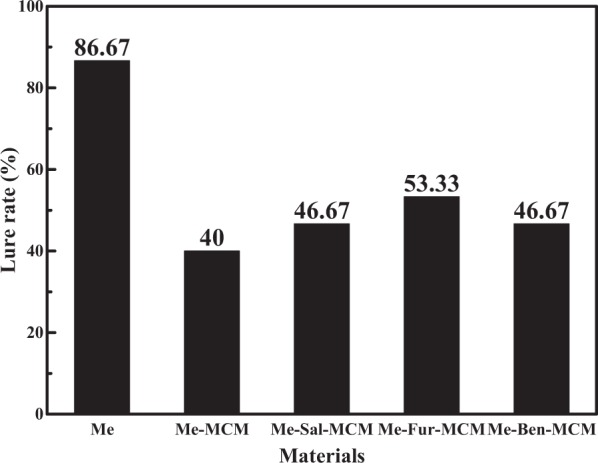
Lure rate of Me, Me-MCM, Me-Sal-MCM, Me-Fur-MCM and Me-Ben-MCM.

The practical performance of the samples was evaluated by attraction of Bactrocera Dorsalis test. The lure percentages of Me-MCM, Me-Sal-MCM, Me-Fur-MCM and Me-Ben-MCM for Bactrocera Dorsalis were 40.00%, 46.67%, 53.33% and 46.67% after 72 h as depicted in Fig. 10. However, pure Me has highest lure percentage (86.67%) due to the decrease in drugs amount actually used in the experiment. While calculated from the TG results for the lure efficiency in one unit drug from Me, Me-MCM, Me-Sal-MCM, Me-Fur-MCM and Me-Ben-MCM have the equivalent of 59%, 69%, 73% and 63% pure Me lure efficiency respectively. In other words, the highest lure rate of the modified MCM (Me-Fur-MCM) equals to the 73% of the pure Me. In summary, the sustained release system get rid of the organic solution and increases the Me service life without affecting its performance significantly.

**Figure 11:**
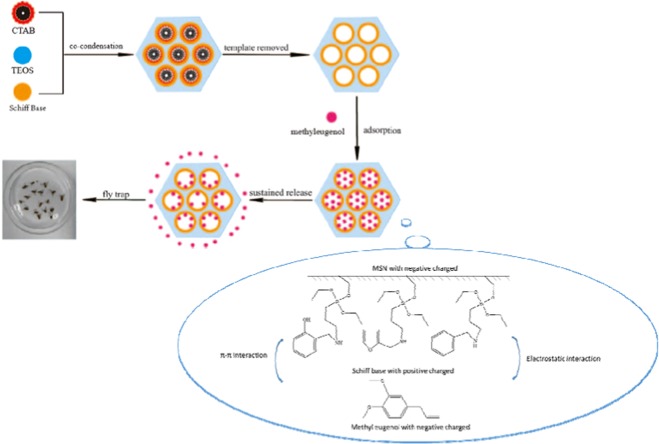
The schematic diagram of drug release of Schiff base modified MCM.

As depicted in Fig. 11, the drug release process of mesoporous silica was illustrated by characterization results and kinetic study in this paper. Schiff base was the link between modified mesoporous silica and Me by π-π interaction between the benzene ring from Me and aromatics from Schiff base and electrostatic interaction between Schiff base with positive charge and Me with negative charge. And the electrostatic interaction played a more important role in interaction between Me and Schiff base modified mesoporous silica. As a result, the release performance of Me was proved and the service life of Me would be significantly prolonged in practical usage.

## Conclusions

In conclusion, Schiff base (salicylaldimine, furfuralimine and benzaldehyde imine) modified mesoporous silica was prepared by co-condensation method. Then Me was post-loaded in the mesoporous silica after removing the templates in the pores. The regular hexagonal pore structure was well-maintained without agglomeration after modification. The existence of interaction between Schiff base and Me was confirmed by the characterization results. And the negative correlation is found between sustained speed of Me and zeta potential of the samples indicating that the electrostatic interaction played a more important role in the interaction between Me and Schiff base modified mesoporous silica. Me is distributed homogeneously in amorphous state in the pores of the mesoporous silica confirmed by DSC results. The loading content of Me-Sal-MCM, Me-Fur-MCM and Me-Ben-MCM 67.89%, 73.34% and 73.84% which was higher than Me-MCM without modification (67.35%). Their sustained release curves could be described by Korsmeyer-Peppas equation in consistence with non-Fickian diffusion mechanism after Schiff base modification. Finally, the attraction of Bactrocera Dorsalis test showed the highest lure rate of the modified MCM (Me-Fur-MCM) equals to the 73% of the pure Me. In short, this sustained release system can avoid the usage of organic solution for dissolution and prolonged its service life without affecting its performance significantly through the enhancement of interaction between drug and carrier through Schiff base modification.

## Data Availability

The data generated or analyzed during the current study are available from corresponding author upon reasonable request.
